# Rethinking Disasters Through Slowness: The Brahmaputra Valley’s Unfolding Transformation

**DOI:** 10.4102/jamba.v16i1.1671

**Published:** 2024-07-18

**Authors:** Kaniska Singh

**Affiliations:** 1 Ashank Desai Centre for Policy Studies, Indian Institute of Technology, Mumbai, India

The book analyses the socio-ecological transformation of the world’s largest river island, Majuli of Brahmaputra River in India. It provokes the reader to challenge the narrow conceptualisation of disasters as spectacular and disjointed episodes of (large-scale) uncertainty, loss, and chaos. Baruah argues that disasters are also constructed through slow, non-spectacular processes, often invisible in the mainstream understanding of disasters. Premised on the everyday experiences of vulnerability, agency, loss, and resistance of riverine societies facing socio-ecological transformation, the book builds on an emerging conceptual lens of ‘slow disasters’.

Anthropological account of the communities and Baruah’s unique dual vantage point – as researcher and island inhabitant – enriches the study by weaving his personal experiences (across chapters) into the intricate tapestry of ethnographic fieldwork conducted across multiple *Chaporis* (small river island) villages. Combining data and perspective offers a nuanced and multi-dimensional understanding of the island’s complex realities. The use of maps, pictures, and colloquial terms in the book helps the reader envision the riverine life and geographies of *Majuli*.

The book offers a political-ecological account of the islands’ transformation into a hazardscape (Mustafa 2005). Through six thematically arranged chapters, it investigates the states’ role in hazardscape making, the shifting canvas of the riverine communities’ livelihood, and their resistance to state interventions.

## Socio-ecological transformation, habitability, and state

The first chapter establishes the book’s intellectual and theoretical landscape. It introduces the research question, theoretical backdrop, the author’s positionality, and the subject of inquiry. In Chapter 2, the vivid descriptions of the reproduction of the Majuli landscape and subsequent socio-environmental transformation shaped by epochal floods and earthquakes in the past and non-spectacular and recurrent flooding and erosion processes paint a grim picture of the shrinking landscape, displacement, and outmigration crisis within the riverine communities. The chapter will push the readers’ engagement on the crisis of habitability in these fluid landscapes (Cons 2013) for riverine societies, especially against the lack of state capacity to fully rehabilitate displaced households and the continuous unfolding of the slow disaster. This transformation is amplified by the colonial and post-colonial state’s prolonged flood-control infrastructural interventions such as embankments, dikes, and porcupines, as argued in the next chapter (3).

The author’s evocative statement, *‘Much of this fetish for hydraulic engineering…is simply a mechanical, repetitive act characteristic of bureaucracy’* (p. 81), cuts through the veneer of progress, exposing the potential redundancy and inertia often embedded within state machinery. While claiming to move beyond the lens of colonial capital domination and *exploitation of nature* to dissect the *disastrous state*, Baruah’s commendable effort falters when entirely unpacking the nuances of the ‘everyday non-unitary state’ (p. 68). The local political dynamics and internal workings surrounding flood control projects remain rudimentary, with sparse evidence leaving this crucial aspect underdeveloped. This lacuna weakens the analysis and may leave readers yearning for a more intricate, textured understanding of the local statecraft. Finally, the current ordering of this chapter disrupts the logical flow of the ‘social’ dimensions of transformation, fragmented across Chapters 2 and 4. Repositioning the chapter after the discussion on livelihood can create coherence and thematic clarity for the readers.

## Unveiling agency and resistance in a precarious landscape

The following two chapters (4 and 5) contain the book’s meat and offer a fertile space for the reader to engage with the interrelationship between the structural barriers to survival in socio-ecological transformation and people’s agency. The meticulous chronicling of the slow and incremental metamorphosis of traditional livelihood practices and livelihood diversification in Chapter 4 showcases the riverine communities’ economic dexterity and ingenious survival strategies amidst the island’s socio-ecological upheaval. The author’s effort to expose the uneven burden of precarious livelihoods on marginalised groups with historical caste and gender divisions is welcome and adds girth to the discussion. Unfortunately, with sporadic and shallow engagement with caste and gender relations, the discussion felt inadequate. For instance, the chapter’s discussion of the gendered labour division fell short of discussing its implications for gendered vulnerability and risk to the slow disaster. Finally, the gender-blind analysis of the anecdotal evidence of participants in the book felt like a missed opportunity for deeper analysis.

The last empirical chapter (5) moves towards local resistance and community response. It offers a critical analysis of the communities’ repertoire of tactics – their extent, array, modus operandi, and even collaborations with local organisations – all deployed against the perceived state indolence in addressing floods and erosion. Interestingly, the chapter reveals a paradox: local demands oscillate between seeking timely completion of anti-erosion measures and simultaneously resisting state-led interventions. This under-explored contradiction holds immense potential for unravelling the complexities of riverine life and the vulnerability production. This chapter ends with problematising the absence of an *island-wide mass movement* (p. 134) and tentatively credits it to the peculiar island geographies imposing certain limitations to mobility. Some discussions in the book (such as the national elections and reduced civil society engagement) are briefly included, leaving the reader confused regarding their relevance to the overall argument.

The final chapter culminates the evidence to situate the state’s flood control infrastructures and negligence ‘at the heart of the slow disaster’ (p. 145) on the riverine island. Complicating the state’s role, Baruah discusses the pull of development discourses, oft-employed by populist governments, pushing for more infrastructures in the fragile islands. Finally, Baruah urges the reader to ‘think like a *Chapori*’. This philosophy calls for *accepting the island’s inherent fragility, respecting the fluidity of riverine landscapes and living in harmony with the Brahmaputra* (p. 149) and *being mindful of social justice and equity*. While this proposition holds merit in its emphasis on respecting the island’s fragility and living in harmony with the river, its direction and effectiveness remain unclear. Readers are left to question whom this proposition targets and how ‘*Chapori* thinking’ addresses the root causes of Majuli’s vulnerability.

Furthermore, this philosophical approach overlooks critical realities. For instance, Majuli’s youths’ increasing aspirations for relocation, or the gendered and casted (caste-based) burden of socio-ecological transformation discussed in Chapter 4, highlights the complexity of adapting to a fragile landscape. The present evidence casts doubt on whether *Chapori* wisdom alone can achieve equity and justice and seems too simplistic. More nuanced analysis is needed to bridge the gap between this philosophical ideal and the messy realities of human needs, ecological limitations, and the yearning for a secure future.

## Rethinking disaster through ‘slowness’

This book valuably contributes to disaster studies by centring communities’ experiences in its conceptualisation of ‘slow disasters’: a much-needed impetus within disaster studies. By conceptualising disasters beyond spatiotemporal concentration, the book brings historical and everyday processes to the forefront, urging a re-examination of disaster frameworks. The ‘unspectacular’ unfolding of Majuli’s crisis, as Baruah notes (p. 9), *underscores the normalisation of compounding challenges faced by riverine communities, often overlooked due to their nature*. Dislodging the focus from spectacular events, the book highlights the slow construction of disasters – a concept built upon Glantz’s ‘Creeping Environmental Problems’ and Blaikie’s ‘slow-onset disasters’. This framework challenges traditional understandings of disasters as singular, dramatic events, instead emphasises non-linear, threshold-independent processes embedded within everyday living. This book’s attempt to make visible the crisis that congregates over a long period, often with slight and silent losses, resonates with the more prominent call for critical disaster studies.

However, the lack of engagement of the book with existing theoretical frameworks such as Knowles’ (2014) ‘slow disaster’ and Anderson et al.’s (2020) ‘slow emergencies’ was surprising. Drawing parallels and divergences from these established concepts would strengthen the analysis of Majuli’s unique socio-ecological transformation and its implications for long-term habitability.

Despite some limitations, the book remains valuable for environmental anthropologists, disaster studies scholars, and those concerned with South Asian riverine communities. It can be a worthy resource for undergraduate and post-graduate geography, disaster studies, and environmental anthropology courses. The book can be a good primer on the South Asian riverplains shaped predominantly by the state’s flood-control infrastructures, providing an overview of the riverine communities’ challenges. The anthropological account of the island transformation is also an extension of scholarly engagement in South Asia’s flood management politics. The lack of depth in some themes is partially overcome by ample referencing and notes at the end of each chapter to complement them, which can be helpful for the readers for further engagement.

The book skillfully balances the historicity of the slow disaster and the ongoing, immediate challenges island inhabitants face and navigate. The book narrates a story of a socio-environmental transformation, a broken hydrological cycle in Majuli islands thrown off balance by state-sponsored flood-control infrastructures, culminating in the slow disaster of erosion and flooding. And yet, the book reinstates the agency, survival, and resistance of island inhabitants of the succumbing *Chaporis* towards livelihood and habitation crisis. The book raises crucial ethical questions about the legacy of state-sponsored interventions and their disproportionate impact on marginalised communities, highlighting the tension between engineered solutions and local realities. By offering a nuanced perspective on slow disasters and urging a reevaluation of disaster management approaches, this book is particularly relevant to recent policy discussions in India regarding displacement caused by river bank erosion.
